# Interconnected Cavernous Structure of Bacterial Fruiting Bodies

**DOI:** 10.1371/journal.pcbi.1002850

**Published:** 2012-12-27

**Authors:** Cameron W. Harvey, Huijing Du, Zhiliang Xu, Dale Kaiser, Igor Aranson, Mark Alber

**Affiliations:** 1 Department of Applied and Computational Mathematics and Statistics, University of Notre Dame, Notre Dame, Indiana, United States of America; 2 Department of Physics, University of Notre Dame, Notre Dame, Indiana, United States of America; 3 Department of Biochemistry, Stanford University, Stanford, California, United States of America; 4 Material Science Division, Argonne National Lab, Argonne, Illinois, United States of America; 5 Department of Engineering Sciences and Applied Mathematics, Northwestern University, Evanston, Illinois, United States of America; 6 Department of Medicine, Indiana University School of Medicine, Indianapolis, Indiana, United States of America; Princeton University, United States of America

## Abstract

The formation of spore-filled fruiting bodies by myxobacteria is a fascinating case of multicellular self-organization by bacteria. The organization of *Myxococcus xanthus* into fruiting bodies has long been studied not only as an important example of collective motion of bacteria, but also as a simplified model for developmental morphogenesis. Sporulation within the nascent fruiting body requires signaling between moving cells in order that the rod-shaped self-propelled cells differentiate into spores at the appropriate time. Probing the three-dimensional structure of myxobacteria fruiting bodies has previously presented a challenge due to limitations of different imaging methods. A new technique using Infrared Optical Coherence Tomography (OCT) revealed previously unknown details of the internal structure of *M. xanthus* fruiting bodies consisting of interconnected pockets of relative high and low spore density regions. To make sense of the experimentally observed structure, modeling and computer simulations were used to test a hypothesized mechanism that could produce high-density pockets of spores. The mechanism consists of self-propelled cells aligning with each other and signaling by end-to-end contact to coordinate the process of differentiation resulting in a pattern of clusters observed in the experiment. The integration of novel OCT experimental techniques with computational simulations can provide new insight into the mechanisms that can give rise to the pattern formation seen in other biological systems such as dictyostelids, social amoeba known to form multicellular aggregates observed as slugs under starvation conditions.

## Author Summary

Understanding bacteria self-organization is an active area of research with broad implications in both microbiology and developmental biology. *Myxococcus xanthus* undergoes multicellular aggregation and differentiation under starvation and is widely used as a model organism for studying bacteria self-organization. In this paper, we present the findings of an innovative non-invasive experimental technique that reveals a heterogeneous structure of the fruiting body not seen in earlier studies. Insight into the biological mechanism for these observed patterns is gained by integrating experiments with biologically relevant computational simulations. The simulations show that a novel mechanism requiring cell alignment, signaling and steric interactions can explain the pockets of spore clusters observed experimentally in the fruiting bodies of *M. xanthus*.

## Introduction

The organization of *Myxococcus xanthus*, the most studied species of the myxobacteria, into structures known as fruiting bodies has long been studied not only as an example of collective motion of bacteria, but also as a simplified model for developmental morphogenesis [1], [2]. Individual *M. xanthus* cells do not have flagella and move on a substrate using gliding motility [3], [4]. The fruiting body process begins when myxobacteria are starved for nutrients and, in response, the population of cells gather into large aggregates containing hundreds of thousands of cells that continue to move around within the aggregate. Eventually, the cells differentiate from motile rod shaped cells to non-motile spherical spores that can wait out the harsh conditions. During this process, a 17 kD protein known as C-signal is transferred between cells and critical to the differentiation process [5], [6]. It has been shown that C-signal requires end-to-end alignment [5], that C-signaling requires cells to move[7], and that C-signal accumulates on cells throughout development process and peaks when spores form [8].

Although the nascent fruiting body contains on the order of 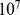 cells, only 1% of the cells in a fruiting body become viable spores [9]. The remaining cells, which constitute the bulk volume of the fruiting body, fail to become spores, lyse, and their extracellular material, polysaccharides in particular, is somehow integrated into the internal structure of the fruiting body. Part of the cell debris would serve as a source of nutrients for cells moving in the mound. Despite the fact that Scanning Electron Microscope (SEM) images showed what appeared as a dense homogeneous packing of spores [9], it is difficult to resolve such a homogeneous distribution of spores with the fact that a bulk of the cells never become spores.

We present, in this paper, an integrative approach that combines a new experimental technique using infra-red optical coherence tomography (OCT) with computational models to study the patterns of spores as they form within a fruiting body. Viewing fruiting bodies by this tomography method revealed that regions of high spore concentrations in the fruiting body were surrounded by less dense regions. Based upon the experimental findings, we developed a hypothesis based on the the underlying biology of *M. xanthus* that can explain the pattern without chemotaxis or long-range diffusive chemicals which have been used to explain other types of biological patterns. Our hypothesis is that the basic mechanism behind this patterning is that cells move along slime trails and reverse to improve alignment so they can C-signal. The increase of C-signal is done locally by cells which coordinates the differentiation process in order for spores to form in pockets of clusters throughout the mound. We present an extended description of the hypothesis from the biological viewpoint in the Results section.

To test if the hypothesis is plausible, we developed two separate models that use different degrees of biological detail. In the two models that we present, we focus on the later stage of the fruiting body process when cells have already aggregated in some domain and the sporulation is beginning. The general modeling approach studies how the coordinated self-propelled cell movement and C-signaling can give rise to the spatial patterns of spore clusters observed in experiments.

We begin with a one dimensional (1D) model that tests how jamming and C-signaling generate clustering on a circular track. The second two dimensional (2D) model implements cell shape and movement and utilizes C-signaling that requires end-to-end cell alignment. The simplicity of the 1D model allows us to study a very wide range of parameter values in order to gain insight into the relative importance of two specific aspects of cell clustering — jamming by cells encountering spores and impact of C-signaling. The 2D model is more computationally demanding and cannot explore the same range of parameter values, but instead focuses on adding more biological details such as connecting cell shape and movement with C-signaling that requires alignment. Model simulation results were compared with the experimentally observed clustering of spores. The hypothesized mechanism based on cells aligning and signaling by contact to coordinate sporulation was able to recover the structure of the fruiting body observed in the experiments. In addition to gaining new insight into bacterial fruiting body formation, better understanding of cell self-organization based on cell-cell signaling and interaction is of real importance for developmental biology.

## Materials and Methods

In order to carry out these studies, we developed an apparatus that integrated the motorized stage of a microscope with a stand alone Optical Coherence Tomography (OCT) device and ran fully computerized scans using LabView to control timing of scans and probe position. Then, we developed preprocessing routines in ImageJ to extract planar cross-sections of data that could then be analyzed by additional programs written in Matlab. The analysis programs extracted statistical properties from the three-dimensional (3D) intensity data and also rendered 3D images of the mound.

### Sample Preparation

We used CTT agar plates for both normal and starved growing conditions of *M. xanthus*. CTT-agar plates are made by adding 1.5% agar by weight to a TPM (10 mM Tris pH 8.0, 1 mM 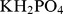, 8 mM 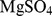) buffer which has 1.0% Casitone by weight. The fruiting body plates are prepared by reducing the amount of Casitone from 1.0% to 0.1%. This results in a starvation condition for the cells growing on the surface and the fruiting body process is carried out. Scans of mound were made 1–2 weeks after inoculating the starvation plates. This is well beyond the 2–3 days needed to form the fruiting body mounds and ensured that the mounds were no longer actively forming.

### OCT Background

In order to examine 3D bacterial density distribution, we employed non-invasive high-resolution infrared optical coherence tomography (OCT) [10]. OCT is an interferometric technique for imaging in scattering media which measures an in-depth profile of optical scattering using light of low coherence. Consequently, a cross-sectional image is created by scanning the beam position laterally over the sample. The fundamentals of OCT relies on the fact that in a scattering medium only the reflected (non-scattered) light is coherent. Correspondingly, an optical interferometer is used to separate scattered light and detect coherent light. A commercially available imaging system (Niris OCT, Imalux Corporation, Cleveland, OH) was used in our work. The time domain OCT system [11] uses common path optical topology, a 1310 nm central wavelength with 55 nm bandwidth, with in-depth resolution of 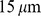 in air and 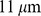 in water. Acquisition time for an image of maximal resolution up to 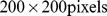 is 1.5 seconds. The OCT probe, mounted on flexible cable, has a diameter of 2.7 mm and a 2 mm lateral field of view with lateral resolution 25 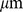. It can be easily mounted in close proximity to the sample to image its full depth (about 1–2 mm above the sample). While the OCT depth-scan is performed by the piezofiber delay line, the lateral OCT scan is performed either by moving the sample or the probe beam illuminating the sample. The OCT was recently used for the analysis of collective motion in suspensions of swimming bacteria [12]. Since the resolution of the OCT is of the order 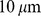, it can only distinguish the large scale structure of the fruiting body, such as cavities and clusters of spores, and not individual bacteria cells.

### Comparison of OCT Method with SEM and LSCM

Scanning electron microscopy (SEM) is a technique that has been used previously to show that spores within a mound are tightly packed [9]. However, preparation a fruiting body for SEM is invasive and requires dehydration in alcohol and other drying agents which likely compressed the structure, removing regions that contain significant amounts of hydrated polysaccharide and extracellular material.

Other researchers concerned with these limitations used laser scanning confocal microscopy (LSCM) with fluorescently labeled bacteria to probe the internal structure of mounds [13]. Preparation for LSCM, like OCT, does not require dehydration so the fruiting bodies can be grown and imaged on agar plates without additional processing. However, LSCM also faces limitations concerning the excitation and emission wavelength of Green Fluorescent Protein (GFP). LSCM typically uses optical wavelength light to excitation a sample and capture the emitted light from a particular focal plain by blocking the out-of-focus light. The use of LSCM to explore fruiting bodies mounds raises the following concerns. Researchers observed that GFP expression appeared to form an outer shell for the dome-like mound. They concluded that the core was likely to be a hollowed out region supported by the extracellular polysaccharide. While they did argue against a differential in GFP expression by cells in the shell and cell in the core, there is another possible explanation for the shell-like pattern.

GFP fluorescence uses 480 nm excitation light and emits at a wavelength of 510 nm [14]. As light travels through any media, it undergoes both Raleigh scattering by particles smaller than the wavelength of the light and Mie scattering by particles that larger than the wavelength of light. Raleigh scattering is inversely proportional to the wavelength raised to the fourth power. This means that excitation light of 480 nm scatters 60× as much as IR light with wavelength equal to 1310 nm, which allow IR light to probe more deeply than visible light. A fruiting body mound is composed of micron-sized spores as well as countless molecules ranging widely over the nanometer scale (most importantly smaller than visible wavelength of GFP). The infrared light used by the OCT provides a better probe for the internal structure of the fruiting body. The trade-off for better scattering depth is the reduce resolution.

In addition to the scattering, *M. xanthus* is known to produce carotenoids designed to absorb visible light [15]. Production of carotenoids is often avoided in lab conditions by growing plates in the dark. Scattering and absorption will determine the maximum depth at which GFP is visible. It was found that the maximum depth for GFP in lung tissue was 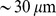 [14]. The average height of mounds in [13] was found to be 27 microns with some mounds reaching heights of 45 microns. It is quite possible that visible light of GFP cannot be detected from the core of the fruiting body. This problem is overcome by using longer wavelength light, like the infrared (IR) light used by OCT.

### Transmitted Light Image

Microscopy was performed on an inverted Olympus microscope and images were taken with a Spot Boost EMCCD 2100 (Diagnostic Instruments Inc.) high sensitivity camera. The camera was still sensitive to the IR probe from the OCT device which appeared as a small white dot in the field of view. This is what enabled the accurate positioning of the probe over specific mounds.

### Scan Acquisition

**Figure 1:**
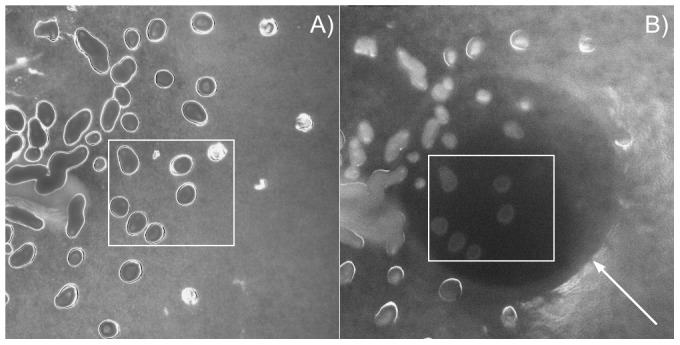
Positioning probe for acquisition. The two boxes highlight same field for the two images. A) 2× magnification of swarm plate without probe. Fruiting bodies appear as dark black circles and ovals with bright perimeters. B) White arrow points to probe head positioned over mounds.

In order to obtain 3D OCT scans of fruiting bodies, we search for a desired region using bright field microscopy at low magnification. Then, using a three-axis micrometer driven translational stage, we position the probe head over the site. The inverted microscope allows for accurately positioning the probe because the mounds can still be seen while the probe head is in the optical path of the microscope (see **figure 1b**). Once positioned, a scan is made by making 2D slices of the mound while the stage is being moved perpendicular to the lateral scan of the probe. The automation was controlled by a LabView program which moved the stage and triggered the OCT for a single slice. The scan parameters could be varied in order to scan a large area of the swarm plate to see many mounds or centered on one particular mound. The distance between slices was usually 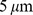. The Imalux Imaging system can be set so that the detected signal for a particular bin is averaged over multiple cycles. This is analogous to a longer dwell time per pixel in Laser scanning microscopy. Averaging was typically done for 20 cycles. There is a trade off between resolution and scan period. The depth of scan also affects the scan period.

### Image Analysis and Rendering

In order to analyze the 3D OCT intensity scan, the 2D slices are loaded into Matlab as a 3D matrix. The raw data is a Red-Green-Blue (RGB)-value image that is converted to an 8-bit grayscale image with an intensity range between 0 and 255. For the OCT scans, the largest intensity values observed were between 180 and 190. Towards the perimeter of each cross section, the values drop to below 10 corresponding to the surface of the mound. There are interior regions where the intensity values reach as low as 80. In-plane cross-sections were extracted from the 3D data by fixing the z-value to obtain a 2D image in the xy-plane parallel to the agar surface. For each in-plane cross section, the image moments 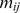 and central image moments 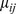 are given by
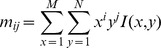


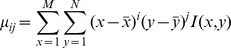
where 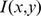 is the grayscale intensity data for the cross section that has 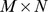 (column
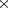
row) pixels. The centroid for the cross-section is given by 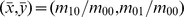. From the centroid, the second central image moments can be calculated as 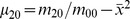, 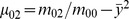, and 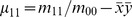. The covariance matrix for the cross section is given by 
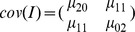
. Finally, the eigenvalues and eigenvectors for 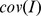 are used to calculate the orientation, eccentricity, and major and minor axes of the in-plane cross section of a mound.

The 3D renderings of the mound are made using the isosurface function in Matlab. The outer shell is an isosurface using an isovalue of 10 and given a large transparency. For the multi-layer isovolume rendering, the highest isovalues which are largely in the interior were rendered with higher opacity. Subsequent lower isovalues were drawn with decreasing opacity so that the internal structure could be visualized.

### Description of Computational Models

To study how the motion of cells and cell-contact signaling within a developing fruiting body could give rise to the patterns characterized by dense pockets showing up as a kind of bumpiness in the OCT, we use computational models that captures the movement of cells in a fruiting body environment. Previously, a 3D Lattice Gas Cellular Automata model was used to study cell aggregation and fruiting body formation as well as spore transport and spatial organization [16], [17].

In both models, we begin simulations with cells in an aggregate and accumulating C-signal. While the vast majority of cells die during the fruiting body process, as evidenced by the fact that 1.0% or less become spores [9], a key importance of cell death is that nutrients are made available to the surviving cells. How the cells die is less pertinent to the current study. The nutrients that come from the dead cells are what allow cells to continue moving and C-signaling in order to reach the level needed for sporulation. We make the assumption that the cells in the models have sufficient energy to maintain their movement. While cell death is not explicit in the model, by enforcing that cells continually move in the aggregate we assume that a source of energy is available. Without enough energy to move, the lack of motility would prevent cells from being able to C-signal [7]. In reality, these energy levels would be maintained by nutrients from the cells in the aggregate that lack sufficient energy and lyse. This approach is used to specifically study how the coordinated cell movement and C-signaling can give rise to the spatial patterns of spore clusters.

#### 1D cells in a track model

Drawing from the expectation that the trajectories of individual cells are confined to the hemisphere of the fruiting body, we developed a zeroth order approximation by modeling cells moving along a 1D circular track. This simplifed model can be used to study the effects of jamming and signaling when cells travel along the same path.

In this 1D track model, cells are initialized randomly throughout the discretized track and move with a constant rate around the track. As cells move along the circular track, they can eventually come to a region that is occupied by spores. This can lead to traffic jams where cells and spores accumulate. In each simulation step, every cell tries to move to the next position in the track. Even though the track is 1D, we do not restrict a position in the track to a single cell. If multiple cells occupy the same position in the track, we say that the cells are side by side in space. This added degree of freedom makes this model a pseudo 2D model. We ignore directional reversals and assume all cells move in same direction. When a cell tries to move into a position that contains spores, a passing probability (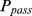) is used to decide whether or not the cell is able to move into that position. 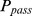 is a number between 0 and 1. A uniformly distributed random number (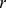) is drawn and a successful pass will occur if 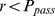. If more than one spore is at a particular location, then the effective passing probability is 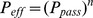, where 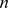 equals the number of spores at the location. Because 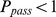, the chances for a cell to pass decreases with more spores.

In this model, motile cells become non-motile spores when they accumulate enough C-signal. To our knowledge, the amount of C-signal required for cells to become spores is unknown. However, in [8], it was shown that the various stages of the fruiting body process were strongly correlated with C-signal levels and that C-signal levels reached a maximum at the point in time when cells became spores. While the regulatory network that causes cells to sporulate is complex, the use of a C-signal threshold for determining when cells becomes spores is a simplification justified by the findings in [8]. In both models we developed, we set an arbitrary value as the threshold for sporulation, which is taken to be 1.0 in this simplified model.

Each cell is also initialized with some amount of C-signal concentration between 0 and 0.5. We assume that C-signal concentration increases by 0.005 each simulation step. Signaling between cells is modeled by a transfer rate (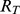) that determines how much signal cells receive from their neighbors. A simplified algorithm used for signal transfer is designed based on the fact that C-signal has been shown to be transferred by cells aligned end to end [5]. To do this, the average C-signal concentration of all cells at a position of the track is calculated. Each cell increases its concentration by the amount 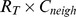, where 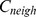 is the average concentration of C-signal for the cells one track position ahead and behind the cell's current position. Cells which occupy the same position do not transfer any signal and no C-signal decrease is applied to any cell. When a cell's C-signal concentration reaches 1.0, the cell becomes a spore and stops moving along the track. 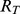 and 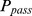 are the two key parameters in this model.

Simulations are run until all cells become spores and then analyzed to study the distribution of spores along the track.

#### 2D stochastic model

In addition to the simplified track model, we extended a 2D stochastic model that was previously developed for the study of myxo swarming in [18], [19]. Because the track model is a minimalistic model, the assumptions can over simplify the particular biological system of interest. We wanted to see whether an extension of the previously designed model that accounted for the shape and movement of cells would recover the patterns of spore clustering observed in experiments. By simulating cells using more biologically realistic movement algorithms in the 2D discs, we studied how the patterns of the in-plane cross-sections from the OCT experiments may have developed.

The features of this model include movement algorithms for *M. xanthus* which uses two motility engines known as A- and S- motility. A-motility is modeled by a cell moving in the direction of its long-axis. S-motility is modeled by cells aligning with cells in a region one-cell length in front of a cell. Additional factors determining a cell's movement are slime trails left by cells and collisions with other cells. In addition, cells undergo regular directional reversals. Cells are represented in the model as three nodes. Each pair of nodes in a cell is connected by a segment that is modeled as a spring and the two segments bend about the middle node. This allows cells to bend and resolve collisions with surrounding cells. (More details on collisions are provided below). One end node of each cell is designated as the head while the other is the tail node. During a directional reversal, the head node and tail node are switched causing the cell direction to reverse. Cells reverse with an average period of 8
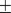
1 minutes based on the experimental measurement of cell reversals in [19]. In this model, the reversal period is independent of C-signal, which is consistent with the description of the Frz proteins acting as the pacemaker of the reversal period as described in [20].

Stochasticity is introduced by adding random contribution to the direction of a cell. The position of each cell is updated by first moving the head node with a constant velocity in a direction determined by the contributing factors — A-motility, S-motility, and slime tracks. A weighted sum of these factors is obtained and normalized to obtain the unit vector pointing in the direction of movement. The weights for each factor is included in **Table S1** in supporting information. After moving the head node, a metropolis algorithm is used to test and accept possible positions of the middle and tail nodes. The acceptance probability for the arrangement of two trailing nodes is determined by the elastic energy 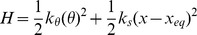, where 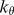 is the bending coefficient, 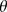 is the angle between segments, 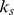 stretching coefficient of each segment and 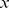 and 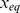 are the current length and rest length of each segment.

The relative strength of the factors determining direction, the reversal frequency, as well as the bending and stretching coefficient are important parameters in the model. The values for these parameters which reliably capture the collective movement of *M. xanthus* cells were established in the previous paper [18], where more complete details for the model are available. Parameter values used in the 2D simulations are given in **Table S1** in supporting information.

Unlike the 1D model where cell-spore interaction is described by a probability, in the 2D model, the cell-spore interaction is determined by the collision process. When a cell tries to move into a position that results in the body of the cell overlaping with the body of another cell, the moving cell attempts to bend and resolve the collision with the other cell. This bending process during cell collisions uses the elastic energy Hamiltonian (H) and metropolis criteria to accept the bent configuration of nodes making up the cell. If a cell's bending is rejected by the metropolis step, then the cell stalls until the next time step. Such stalling is resolved by either a change in positions of the other cells surrounding the stalled cell or by the stalled cell reversing. Similarly, when a cell collides with a spore, it must do this bending procedure in order to get around the spore. Since spores are round instead of rod-shape, the spatial effect of cell-spore collisions is different from the cell-cell collisions. Because the movement of cells determines the cell-spore interaction, there is no need to include a parameter that defines a passing probability like that used in the simplified 1D model.

Because the model was already validated for cell movement, the extensions we made focused on C-signaling and sporulation. In order to extend this model for testing our hypothesis of the mound structure formation, we added components describing the C-signaling and sporulation. This was done by assigning each cell a counter for the C-signal and introducing two cell states: 1) motile cell and 2) non-motile spore. Motile cells accumulate C-signal by contact with other motile cells. Signal transfer between cells requires cells to be aligned and touching end to end. From each cell, we define the orientation as the vector pointing from head to tail and consider cells aligned if the angle between two cells orientation is less than 30 degrees. The polarity of cells does not matter (i.e. contact between two heads, two tails, or a head and a tail all cause signal exchange). As with the track model, side-by-side cells did not exchange signal. At every simulation step, we test to see if two cells match the requirement for signaling and, if so, the two cells exchanged signal. Each signal exchange between two cells causes the C-signal counter of the cells to increment by 1. When a cell reaches a signal threshold of 500, the state of a cell is changed from motile to non-motile and the rod-shape body is replaced by a round circular spore.

Since the amount of C-signal increase is set to one unit per signaling event, the threshold determines the rate of C-signal accumulation. By setting the threshold lower, each C-signal event accounts for a larger increment relative to the threshold. In contrast, setting the threshold higher causes each C-signal event to be a smaller increment. In a simulation where the threshold was set twice as high as the default value of 500, we did not see a significant difference in the spatial patterning despite the additional time needed by cells to become spores.

**Figure 2:**
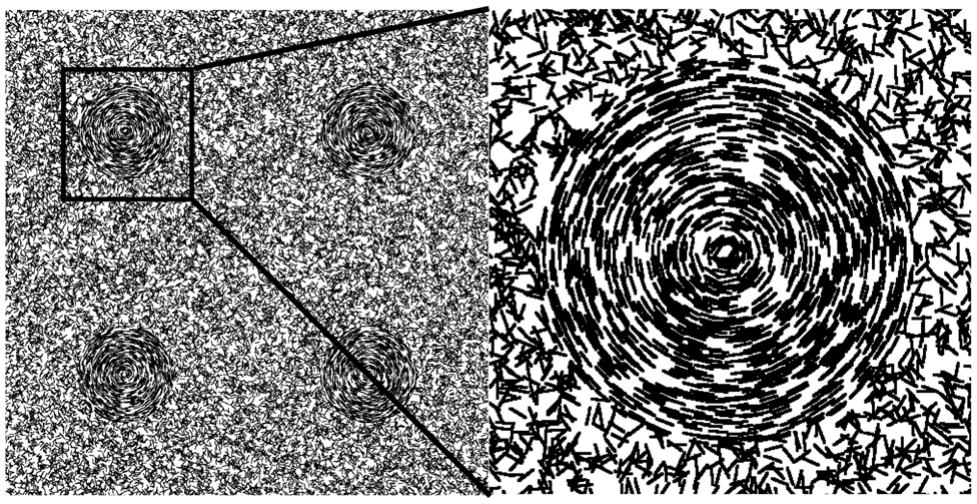
Initial conditions of 2D simulations. All cells are position randomly throughout domain, but cells that fall within the region of the discs have the orientation set tangent to the radius of the disc. Orientation of cells outside disc is random.

In order to simulate the aggregation of a fruiting body, we set up a 500×500 micron area with a periodic boundary containing 4 discs with a radius of 50 microns. Initially, 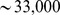 cells are distributed randomly throughout the whole domain. The cells which were randomly placed inside the circumference of a disc were given an orientation tangent to the radius of the disc to set up a rotation consistent with the fruiting body formation (see **figure 2**). To be clear, during the simulation, the movement of cells inside the disc is not confined to a fixed radius or track as in the 1D model, but rather cells move in 2D domain based on local conditions including slime trails laid down by other cells. The slime trails and orientation of neighboring cells reinforces a rotational pattern of movement. At the end of simulations, we analyzed the distribution of spores within the discs and compare the results with the experimental data and the results from the 1D track model.

## Results

### Transmitted IR Light Imaging

**Figure 3:**
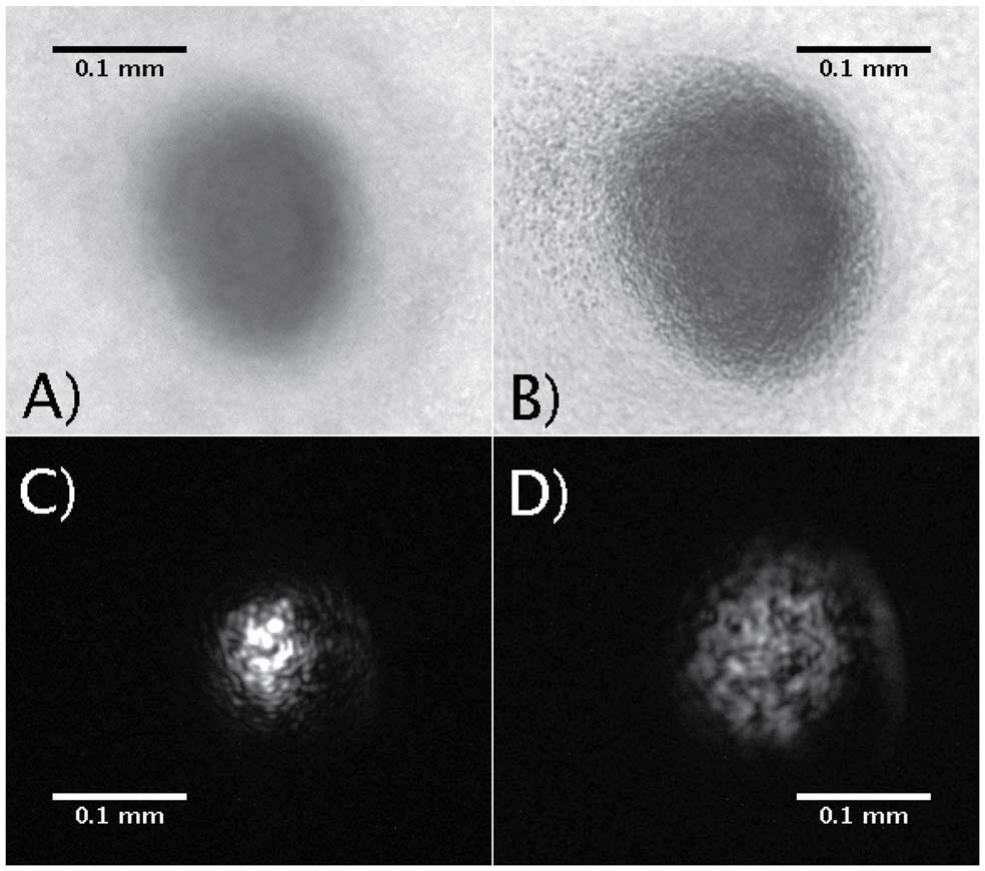
Bright-field transmitted vs infrared transmitted microscopy. The two different modes are shown using two different focal planes. A) and C) are focused near the top of the mound, while B) and D) are focused closer to the surface. The large scale irregularities seen in the OCT scans can be optical observed in C) and D). No internal structure is seen in visual light, panels A) and B).

The use of the OCT method to scan fruiting bodies was expected to accurately reveal the internal structure due to the improved scanning depth of IR light (technical details for this reasoning are made in the Materials and Methods subsection ‘Comparison of OCT Method with SEM and LSCM’). In fact, we suspect the core of the fruiting body mound cannot be probed with visible light. Evidence for this is seen in the dark appearance of mounds in bright field images (see **figure 3**). It was discovered that microscopy images of mound structure could be made by using the IR probe as a transmitted light source for the inverted microscope. We centered the infrared light from the probe over a mound to obtain the images which revealed details not visible in the bright field images of mounds. A side by side comparison of bright field images using optical light and IR light can be seen in **figure 3**. While optical light does not transmit through the mound, the IR light passes through the mound and reveals contours and structure not seen in the bright field images. The IR transmitted light image showed structure that is similar to the structure we find in the 3D renderings of the OCT tomograms (see ‘Large scale inhomogeneous internal structure of fruiting bodies’ below).

### OCT Scans Improved with Oil Immersion

**Figure 4:**
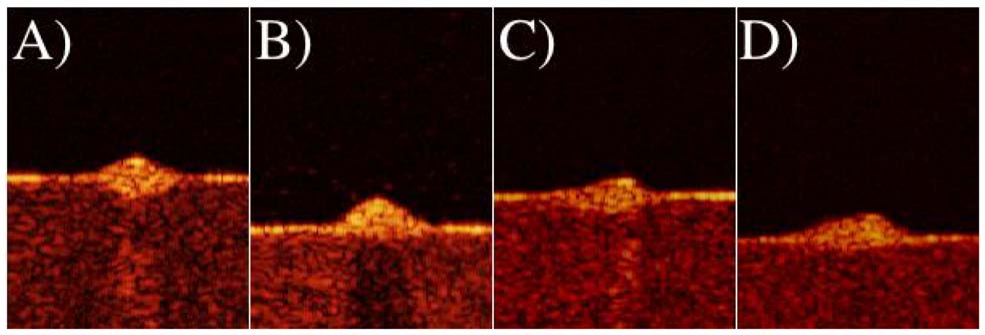
A single slice of an OCT scan for different scan media. The dark region is the space above the surface of the agar plate. The red area is the agar. The yellow area is the mound as well as the bacteria on the surface. A) Dry Scan. B) Oil Immersion Scan of mound in (A). C) Dry Scan. D) Glycerol Immersed Scan of mound in (C). Brighter regions below mounds in (A) and (C) demonstrate lensing effects for dry scans.

Individual slices made by the OCT can be seen in **figure 4**. Early scans suffered from light reflecting back to the probe from the top of the mound. This reflection is problematic because the probe detects an increased signal directly above the mound. Additionally, the reflected light cuts down on the amount of light moving into the mound which reduces the noise to signal ratio. It was also found that dry scans also suffered from a lensing effect due to the change of index of refraction from air to mound. This lensing effect resulted in the OCT instrument detecting higher levels of backscattering underneath the mounds below the surface of the agar. (See bright area below the mounds in **figure 4A and C**).

To improve the quality of image, we place a drop of microscopy oil on the surface of the agar plate and submerged the probe head into the oil. **Figure 4** demonstrates the difference between imaging in air and in oil. We also performed tests with glycerol that showed similar improvement. However, because the oil is immiscible with the water in the agar and does not evaporate, it provided a better submersion media for the scans. The submerged scans produced a better contrast for signal to noise within the mound and cut down on the reflected light. It has been shown that the refractive index for bacteria is 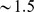 [21]. This explains why microscopy oil, with a refractive index of 1.53, is an ideal submersion media for the bacterial mounds. While scanning in oil, the OCT device corrected for the index of refraction by adjusting the number of pixels used in a scan. This required rescaling the image in the vertical direction to recover the 1∶1 aspect ratio of the scan. Finally, a ratio of 3.3 microns/pixel was adopted.

### Large Scale Inhomogeneous Internal Structure of Fruiting Bodies

The detailed analysis of an OCT scan of a fruiting body mound was carried out to study its internal structure. The image analysis (described in Materials and Methods) was able to quantify both the internal structure and the external shape of the mound.

**Figure 5:**
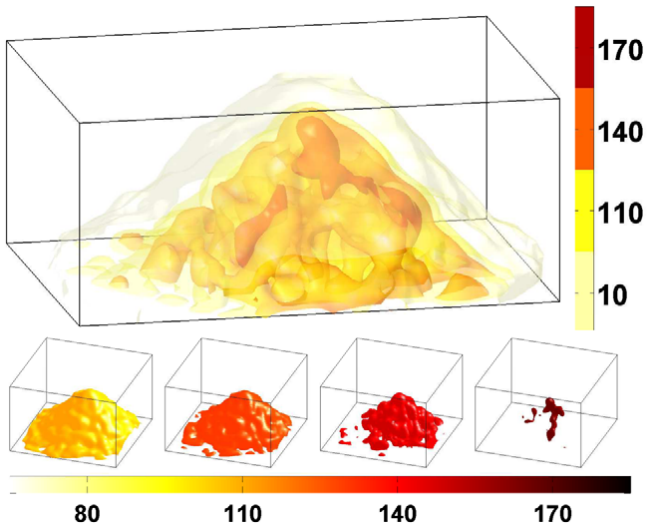
3D rendering of fruiting body density. The top image is a sample mound illustrating a 3D rendering of OCT scan of a mound. The colorbar indicates the intensity value. Higher intensity values corresponds to more back scattered signal which is indicative of regions with higher optical density. In the bottom row, 3D renderings of isovolumes for different intensity values. The intensity value for each isovolume is marked on colorbar. (Lighting and rendering effects causes the color to appear slightly different than the corresponding color in the colorbar).

**Figure 6:**
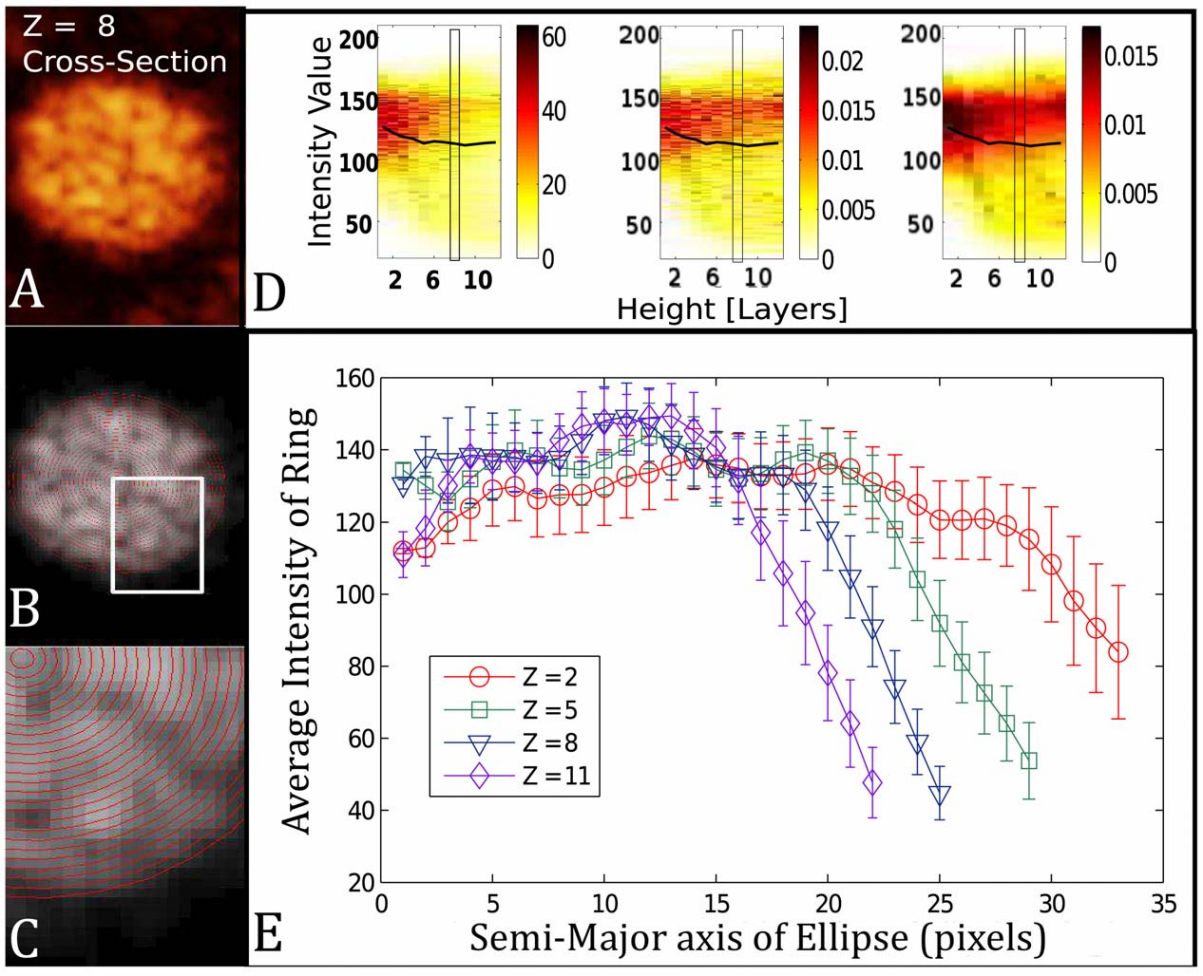
Detailed analysis of OCT scan. Left Column Panel: A 2D rendered cross section from sequence of OCT slices. A) Raw data cross section. B) Gray-Scale image used for image analysis. The elliptical domains for radial density are overlayed on the image. C) Zoomed in region of (B). D) Analysis shows the average intensity (black line) and intensity distribution for cross-sectional discs as a function of height from the base of the mound. The x-axis is the height of each disc in the mound where zero is the base. Data is from mound shown in **figure 5**. The left plot is the unnormalized data that shows how many pixels have a given intensity value. Middle plot shows the distribution within each disc normalized by the area (total pixels) of each disc. Right plot shows the results from smoothing the distribution of each disc. The black rectangle in all three plots corresponds to the distribution for the cross-sectional disc in (A). E) Analysis showing radial density for four of the elliptical discs at different heights within the mound. Data is from the fruiting body shown in **figure 5**. The 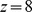 line is from cross-section shown in (B).

**Figure 7:**
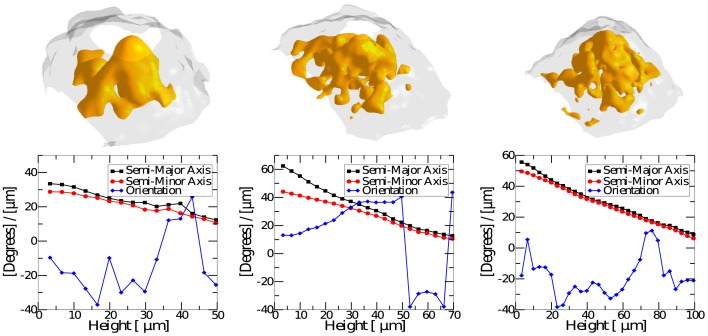
Renderings and shape characteristics of three mounds. The upper row of images shows 3D renderings of three different mounds. For each mound, the lower row of the figure plots semi-major and semi-minor axis for cross sections as a function of the height from the base of the mound. The orientation as a function of height is shown in same plot. The axis plots are in microns while the orientation is in degrees.

Scans show no indications of a shell and core structure that was suggested by the LSCM study [13]. Instead, they reveal a continuous inhomogeneous density structure containing intensity patches that could reflect variations in the spore concentrations. These domains can be seen in both the three-dimensional renderings (**figure 5**) as well as individual in-plane cross-sections (**figure 6A**). In these images, the optical density, measured by intensity, depends on the density of the scatters in the media, i.e. the concentration of spores. Hence, the intensity levels of the OCT scans are proportional to the density of the mound. Each in-plane cross section is analyzed as an elliptical domain whose major and minor axis are obtained from the covariance matrix. The linear decrease of the axes as the height increases is consistent with a cone-like mound (see **figure 7**). The average intensity was obtained for each cross-sectional elliptical domain as well as the radial density. **Figure 6D** shows an example of the intensity distribution for a domain as well as the mean value of the intensity for the cross-section. Radial density plots were obtained for each in-plane cross section by averaging the intensity values for all pixels with in an elliptical annulus (**figures 6B and C**). The standard deviation from the mean value provides a measure for the variation of the density within a particular ring of a particular domain. The radial density for multiple domains from the mound shown in **figure 5** can be seen in **figure 6E**. The regions of reduced density may reflect cavities or lower spore concentrations, while regions of high density are suggestive of closely packed clusters of spores. The graphs in **figure 6D** show the highest density at the base of the mound and a consistent average density up the mound until it begins to taper off towards the top. The distribution of intensity values is shown for bottom 12 layers of the mound (i.e. the in-plane cross sections). The graph in **figure 6E** shows that the average radial density ranges between the values of 120 and 140 for distances up to approximately 15 pixels (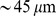) before tapering off. The radial density plots for individual cross-sections reveal the variation that exists within a given elliptical ring (shown as error bars) as well as intensity variations moving radially outwards from the centroid. For the in-plane cross-section 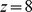 and 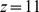, we observed the region of increased intensity of approximately 6 pixels (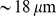) at the distance of 12 pixels.

**Figure 8:**
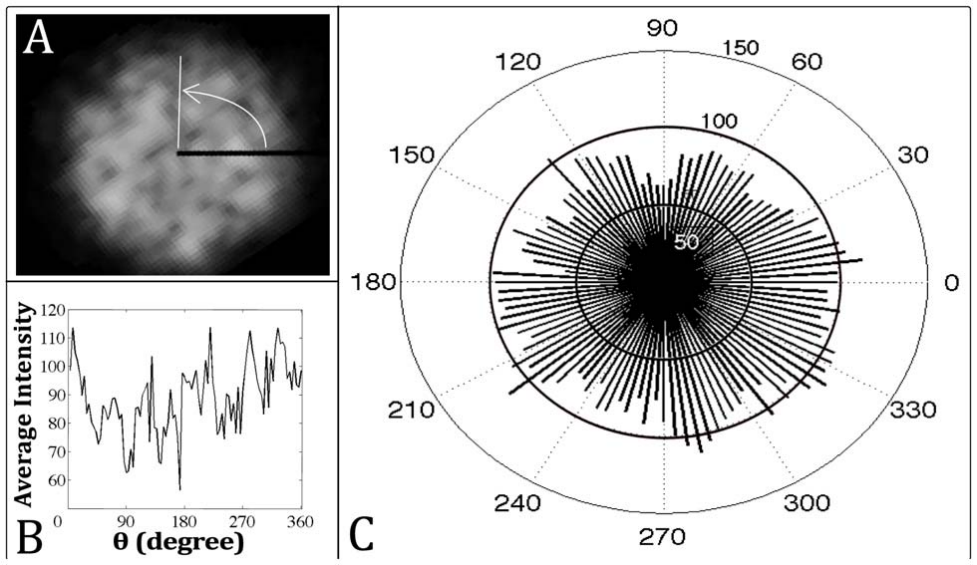
Angular distribution of intensity averages for experimental cross section. A) Image of cross section. The white line shows where the reduced intensity occurs around 90 degrees. B) Intensity distribution as a 2D plot. C) Distribution shown as polar plot.

In addition to the radial distribution, we performed measurements of the angular distribution of intensity. This was done by dividing the domain into sectors (i.e. pie slices) and averaging the intensity within each sector. The results for one cross-section are shown in **figure 8**. The zones of lower concentration are spread out over the domain and is characterized by peaks and valleys in 2D plots of the distribution (**figure 8B**) and a smooth undulation in the polar plot (**figure 8C**) of the distribution. This measurement is repeated in simulations and provides a metric for comparison.

The more striking features that are revealed by the 3D OCT scan are the large interconnected structure of caverns with in the mound. **Movie S1** in supplemental material provides a more complete view of the internal structure. Scans show what appear to be regions on the scale of 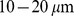 that are more dense than surrounding regions suggesting that large regions of highly packed clusters of spores are spread throughout the mound (see **figures 5** **and** **6A**). The regions surrounding these clusters contain material that is less optically dense than the clusters of spores. This could be regions of polysaccharide and extra-cellular material or simply a region where the spores concentration is reduced.

### Biological Hypothesis for Pockets of Spores Forming in Fruiting Body

The findings from the experiments were used to contemplate the bigger picture of fruiting body formation, which is presented here. In the fruiting body process, a mound of constantly moving cells gives rise, after several days, to a mature, spore-filled fruiting body [22], [23] (see **figure 1**). Inasmuch as growing cells have little ATP or other energy reserve, starvation liberates a tiny minority – 1% or fewer of the rod-shaped cells – to cannibalize the other 99% of the developing cells to harvest enough metabolic energy for developmental protein synthesis and for keeping the cells in constant motion as they develop further towards becoming spores. Collisions between moving cells eventually raises the morphogenetic C-signal to a threshold level that is able to trigger differentiation of rod-shaped motile cells into spherical spores [8], [24]. However, before they sporulate, the developing cells aggregate by moving back and forth in a system of traveling waves that surrounds the swarm edge, a process that has been captured in a time-lapse movie [25]. Like the traveling waves assembled for fruiting body aggregation, very similar waves are observed when *M. xanthus* feeds and grows on *E. coli* prey cells [26], [27]. In fruiting body development, the vast majority of myxobacterial cells are being eaten by their few siblings that are destined to become spores. Although prey protein, nucleic acid, and lipid are consumed for their calories, the polysaccharides are indigestible. The myxobacterial lytic enzymes include no polysaccharide hydrolases except lysozyme [28]. Polysaccharide fibrils [29]–[31] survive in the waves as an extensive elastic meshwork that surrounds and bundles the cells as well as their undigested bits of cellular debris. The few prespore cells that survive are found to be moving on trails of their polysaccharide slime that also is not digested. Trails remain intact and provide a surface favorable for gliding [32].

Departing from the traveling waves, the surviving cells are seen to migrate to the outer edge of each wave crest where they become one of the small motile aggregates [25]. Initially the motile aggregates are spaced one wavelength apart, and waves are thus the first step in fruiting body aggregation. Next, pairs of adjacent small aggregates fuse with each other to form a larger spherical aggregate of the same cell density but twice the volume. The larger aggregates fuse repeatedly with their neighbors until all the motile aggregates have assembled in a single very large aggregate. The diameter of the final aggregate, which shows signs of cell movement inside [25], is constant from experiment to experiment and is characteristic of mature *M. xanthus* fruiting bodies. (See [33], for example.) In this way, spores are expected to be formed on debris-laden slime trails that are suspended within a spherical motile aggregate by polysaccharide fibrils. Each slime trail would be expected to trace a truncated arc within the motile aggregate because each cell reverses direction of its motion at regular intervals [19], [34].

Based on the foregoing description of slime trails in a dynamic motile aggregate, it follows that cells would be clustered and aligned on the many trails that would branch from each other. Since individual cells are eating each other as they move, they are also racing to be one of the predators that survive rather than one of the prey that expire. In such a race, long chains of rod-shaped cells, moving on the same trail, would break into shorter segments of fewer and fewer cells until only 1% — to take some definite number since the number depends on residual nutrient — of starting cells remain on the trails and able to transmit C-signal. When two counter-migrating cells on the same trail collide end-to-end, they exchange C-signal with each other. C-signal transmission continuously raises the signal level in each cells outer membrane through positive feedback and the Act system [8]. Eventually, positive feed-back raises the level of C-signal in each cell to the threshold required to differentiate a rod-shaped cell into a spherical, non-motile, dormant spore [8]. Depending on each cells unique history of C-signaling, individual cells will reach the threshold at different moments. Nevertheless, the closer two cells are found to each other on the same trail, the more correlated their time to reach threshold will be. When a rod-cell becomes a spore, it remains on its debris-laden slime trail, and each trail would form some arc within the aggregate mound. Because most cells are destroyed, there will be many trail arcs each of whose spores will have formed at the same time, while different arcs will have sporulated at different times. Finally, the slime trails collapse around their own cluster of spores. As this nascent fruiting body dries out, the polysaccharides will also lose water and the aggregate will shrink. Within the fruiting body, the spores are likely to be clustered in space on their own arc-shaped trail that collapses into a ball of spores and polysaccharides.

### Simulation Results

To test the hypothesis described in the previous section, we ran simulations with the two models we developed.

#### 1D track model results

**Figure 9:**
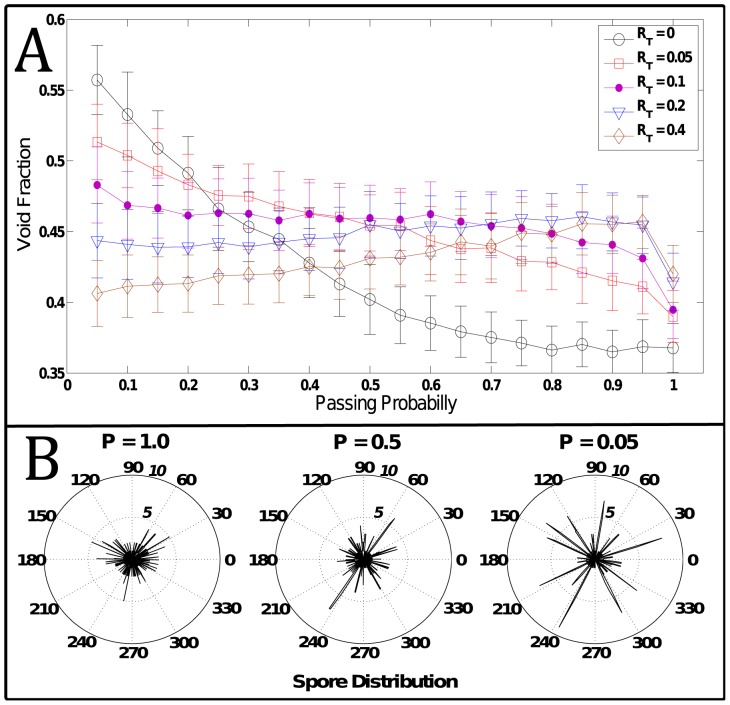
Simulation results for 1D cells in track model. A) Plot shows the effect of decreasing the passing probability from 1.0 to 0.1 by looking at the fraction of empty positions in the track. The different curves correspond to different rates of C-signal transfer between neighbors. Results are shown for 20 different values of passing probability and five different transfer rates. Averages were taken from 100 simulations for each set of parameters. B) Spore distribution along circular track for three simulation results. The radial distance corresponds to the number of spores while the angular position denotes the position in the circular track (The outer circle is for a spore count of 10 while the middle circle corresponds to five spores). The passing probability P varies for the three simulations from 1.0 to 0.05. Figure demonstrates how spores clustering is increased as passing probability decreases.

One advantage of the 1D model is the ability to explore a large parameter space due to the low computational demand. To study the impact of the passing probability and rates of signal transfer, simulations were run for a range of passing probability from 0.05 to 1.0 and a range of 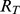 from 0 to 0.4. The passing probability range was selected to cover a range from cells always passing spores to cells almost never passing spores. This range studies the general behavior of the parameter even though a biologically plausible value likely exists in a subset of this region. For 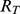, the range of values included showed a noticeable change in behavior while simulations with 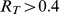 showed similar behavior to 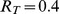 and were excluded from the data presented. One hundred simulations were run for each parameter set and the results are given in **figure 9**.

It should be pointed out that the passing probability parameter in the 1D model is a simplification of the cell-spore interaction and does not attempt to define the nature of the cell-spore interaction. The impedance of a spore to a passing cell could be adhesive, spatial, or some combination of both. The parameter only defines how much impedance a spore presents to a moving cell. However, in the 2D model, we specify the cell-spore interaction as collisions with no adhesive interaction (see section “2d Stochastic Model”).

An example of how spores are distributed along the circular track at the end of several simulations is given in **figure 9B**. The pockets of spores interspersed by voids (i.e. empty track positions) can be seen throughout the circular track. The graph in **figure 9A** represents the average void fraction (i.e. fraction of the track without any spores) as a function of the passing probability. The different curves correspond to the different levels of 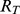.

For this range of passing probabilities and 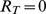, a 50% increase in the void fraction was observed. When signal transfer is introduced (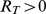) an interesting interplay develops between signaling and jamming. From **figure 9A** we see how the void fraction (i.e. clustering) depends on the passing probability. This dependence changes with C-signal transfer rates. With no signal exchange between cells and only a basil production, C-signal concentration for a cell increases linearly in time. After turning on signal exchange, the C-signal concentration dynamics becomes a non-linear process that depends on the concentration of C-signal for the neighboring cells. For the 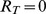 curve, the decreasing void fraction is not a linear effect as seen by the rate of decrease being large at lower passing probabilities but small for high passing probabilities. The simulation sets with C-signal transfer exhibit a linear dependence between void fraction and passing probability (excluding the value of 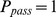 where all void fraction values drop). Particularly interesting is that the level void fraction changes from decreasing for low transfer rates to increasing for high transfer rates. This complex behavior can be attributed to the non-linear effects of the C-signal model. This behavior suggests that this contact-based signaling can actually regulate the degree of the spatial clustering over a range of passing probability values.

We have the following explanation for the fact that high jamming (i.e. low passing probabilities) and larger signal transfer rates lead to less clustering (i.e. smaller void fractions). By increasing the C-signal transfer rate in the model, cells will reach the threshold for sporulation more quickly. Because C-signal molecules need to bind to a receptor on another cell, an increase in the C-signal transfer rate is analogous to the binding between C-signal molecules and receptors occurring more easily. The faster cells become spores, the less likely it is for them to move to a point on the track containing a cluster of spores. In contrast, smaller transfer rates allows a cell to reach a cluster on the track, become stuck, and then change into a spore. In the range of higher passing probabilities (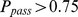) where cells do not jam as easily, the rate of C-signal transfer has the reverse effect on the void fraction. In the absence of strong jamming effects, contact-based signaling is seen to partially recover the clustering of high jamming simulations. From the simplified 1D track model, we can see how both impedance due to cell collision as well as cell signaling can lead to the pattern of spore clustering.

It is interesting to note that jamming in the absence of signaling (i.e. 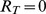) does not produce substantial clustering until the passing probability drops below 50%. This is significant because observations from experimental movies show cells resolving head-on collisions smoothly without stalling. Also, cells can pass through by squeezing between cells moving as a cluster in the opposing direction. These observations suggest that there should be large passing probabilities for cells moving inside the fruiting body where the influence of jamming is limited.

The aggregation sites of spores are the discrete positions on the track where clusters of 5 – 10 cells can accumulate as seen in **figure 9B**. Since these sites in the model are collection of point like particles, we cannot directly compare the shapes of these simulated clusters with the clusters seen in the in-plane cross-sections from experimental data. Hence, the 2D model can improve upon the general observations we gain from the 1D model.

#### 2D stochastic model results

**Figure 10:**
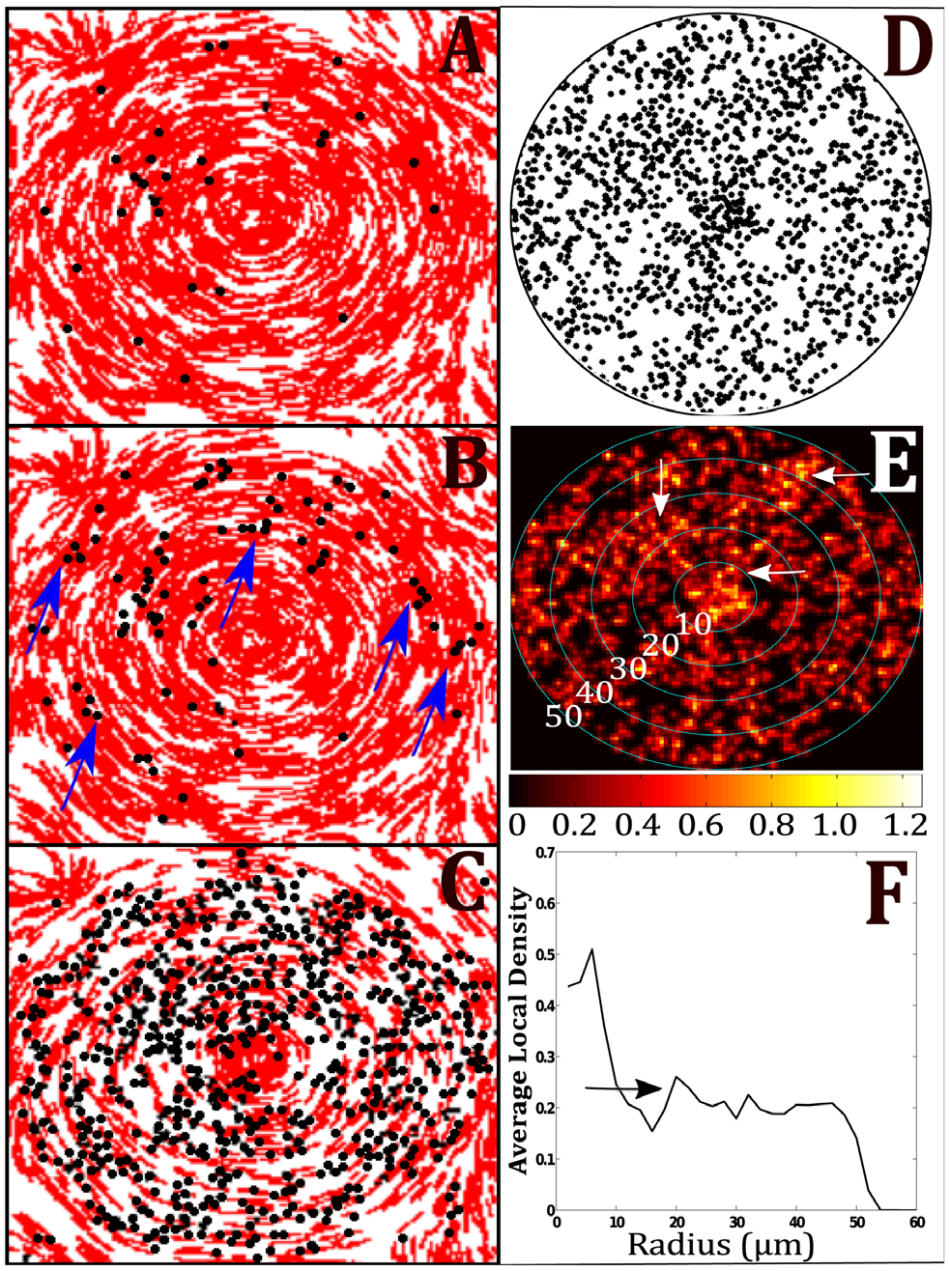
Agent-based model results. A–C) Snapshots from simulation over time. Frame (A) shows the initial locations of the first few spores within the mound. Blue arrows in (B) point to regions where groups of two and three spores form together. (C) Later stage of simulation shows the distribution of spores filling in around the disc. D) Spore positions in disc at end of simulation. Radius of disc is 50 microns. E) Local density field for simulation results in (D). Circles show several radii values for radial local density. F) Radial local density distribution for (E).

While some general observations on the level of clustering can be drawn from the 1D track model, simulations using the 2D stochastic model provide insight into how the coordinated movement of cells and alignment-dependent signaling lead to the patterns of spore clusters. During the simulation, the movement of cells inside the disc is rotational due to the cells aligning with their local neighbors and the slime trails that are set up by other cells in the disc. In **figure 10A–C**, a sequence from the simulation demonstrates the rotational movement of cells inside the disc and the positions of spore formation inside the aggregate. Cells that collide end-to-end and are aligned accumulate C-signal until they reach the threshold for sporulation. The cells whose movement within the aggregate results in the most aligned contact with other cells are the first to become spores. **Figure 10A** shows how the first several spores to form are arranged within the mound. While some spores are isolated, there are several regions containing two and three spores in close proximity. **Figure 10B** shows how several new clusters of two to three spores appear within 25 simulation steps of previous frame. (Blue arrows in **figure 10B** designate several of the clusters). This demonstrates how the contact-dependent signaling by cells coordinates the differentiation process. In the last image of the sequence (**figure 10C**), the spores are seen to form in clusters of spores throughout the mound with few cases of isolated spores. **Figure 10D** shows the positions of spores at the end of the simulation.

In order to compare with experimental data, we determined the local density of spores inside a disc from our simulations. A square region of 400×400 pixels containing a disc was divided into a 100×100 rectangular grid. The number of spores contained within each grid point inside the disc was used to determine the concentration field. A local density field was created by convolving the concentration field with a gaussian filter. By using the gaussian filter at each grid point of the concentration field, we calculated a weighted average of spore concentration for each grid point. This averaging is similar to the measurements made by the OCT device due to the finite diameter of the scanning beam.

**Figures 10D** and **E** show the positions of spores at the end of a simulation and the corresponding local density field. By taking the average overall all grid points in the local density field at a fixed radius, we obtained the radial distribution shown in **figure 10F**. Three large zones of high cluster density are pointed out to by arrows in **figure 10E**. Small dark bands corresponding to low spore density surround these high density zones. By comparing the simulation local density field with the in-plane cross-sections from the experimental data, we see there is a good agreement between simulations and experiments. In both, we see cavities of low cell density form throughout the mound and surround large pockets of higher density. The radial distribution shows that the simulated 2D fruiting body has a radial density distribution that peaks near the center but then falls quickly to a value that is consistent out to the edge of the disc. The bump in the radial distribution at 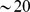 microns (see arrow in **figure 10F**) is consistent with the patch of higher concentration to the left of the disc center highlighted by the vertical arrow. We can see that the thickness of the bump is about 15 microns which is consistent with the scale of the high density regions in the experimental data.

**Figure 11:**
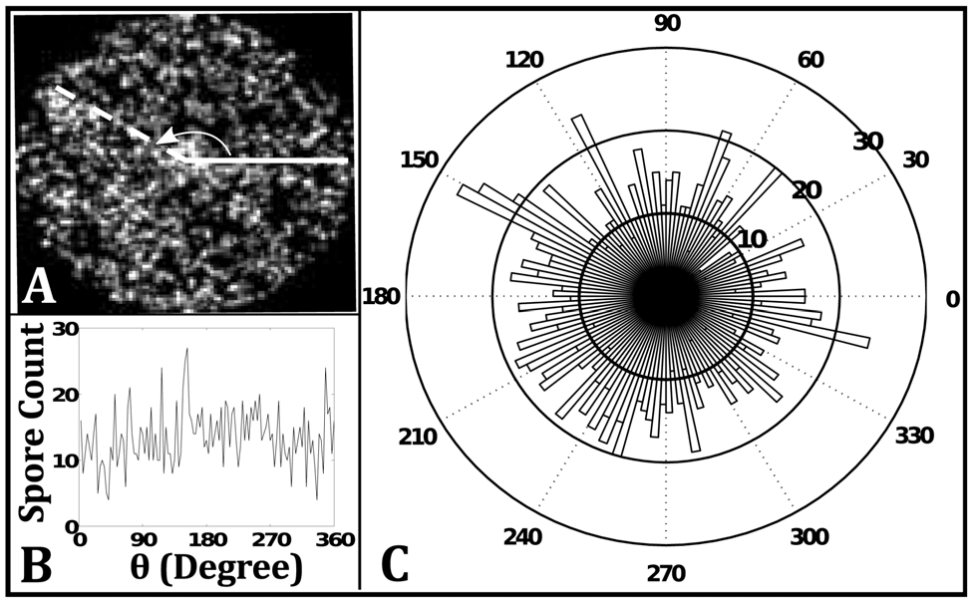
Angular distribution of spores for simulated disc. A) Local density map for 2D simulation. The dashed white line shows where the reduced spore concentration occurs next to high concentration pocket around 150 degrees. B) Concentration of spores in sector of disc as a 2D plot. C) Concentration shown as polar plot.

To compare with the experimental angular distribution, we performed an analogous measurement on the simulation data. From the concentration map, an angular distribution of spores is obtained by summing up the number of spores in grid points that fall within the same three degree sector of the disc (i.e. a pie slice). Results for this measurement are shown in **figure 11**. The image in **figure 11A** is a gray-scale version of the local density field (**figure 10E**), but rotated by 90 degrees for easier comparison with the polar plot of the angular distribution in **figure 11C**. The angular distribution shows qualitatively similar undulation as the experimental data. The dashed line in **figure 11A** highlights the regions around 150 degrees in the distribution plots that shows high levels of spores next to the low level of spores between 120 and 150 degrees.

## Discussion

A novel imaging technique – infrared optical coherence tomography – revealed that hundreds of thousands of spores in a mature fruiting body of *M. xanthus* were not packed uniformly, as was surmised previously. Rather, the spores are found clustered in high density pockets, which are no larger than 25 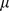
m in diameter, that are separated from each other by domains that have reduced concentration of spores. Why should the fruiting bodies have numerous cavities with relatively fewer spores? Detailed analysis of the way that fruiting bodies form based on the experimental observation of dense pockets yielded a biological hypothesis of how the movement, alignment and C-signaling of self-propelled rod-shaped cells could coordinate the differentiation process (presented above in “Biological Hypothesis”). In what follows, we discuss the outcome of the computer simulations designed to test the mechanism of the fruiting body formation proposed in the biological hypothesis.

First, our 1D track model provided two possible explanations for the formation of the cavernous structures of the fruiting bodies. One explanation was that early sites of spore formation act as focal regions for spore clusters due to jamming of the motile rod-shaped cells that continued to move around the track. This explanation suggested that high levels of clustering could result from spores strongly inhibiting the motility of cells. The highest level of clustering was observed when cells had the smallest passing probability and no C-signal transfer. However, in simulations with higher passing probabilities, (i.e. motile cells were not strongly inhibited by spores), more clustering was seen when C-signal exchange by local cells was present than when only jamming was considered. Experimental movies from our previous study on cell-cell collisions [35] demonstrate the flexibility of myxo cells which would allow easy resolution of collisions with spores. This indicates that having a higher passing probability in the 1D model is more biologically realistic. A mechanism of only low passing probability when spores strongly inhibit cell motility but cells do not signal is not strongly supported by the experimental observations. Rather, the 1D model shows that C-signaling can increase the level of clustering in simulations with higher passing probabilities.

The 1D model simulations initially confirmed that contact-based C-signaling would generate spore clusters when the cell-spore interaction was not characterized by strong spatial jamming. These findings were the motivation for focusing on the movement and alignment of cells in a more detailed model. Thus, the 2D model was used which could account for the biological details such as cell-shape, movement, and alignment-dependent C-signaling. The 2D model simulations have shown how the patterns of spore clusters could be produced by cells moving, aligning and C-signaling to coordinate differentiation. In the simulations, spores begin to form within a disc as small clumps (see **figure 10B**). The reversals of cells within the disc cause them to move back and forth along specific trajectories or arcs within the fruiting body. Cells that spend time moving along the same trajectories in end-to-end alignment accumulate C-signal at similar rates. This leads to spores forming in clusters throughout the mound. The simulations we performed to test the hypothetical mechanism resulted in pattern formation consistent with the experimental data. (Compare **figure 10E** with **figure 6A**).

To summarize, we first formulated a hypothesis based upon the experimental observation of spore patterns in fruiting bodies. We hypothesized that pockets of dense regions of spores form because cell movement, alignment and signaling result in coordination of the cell differentiation. The 1D simulations demonstrated that cell-signaling was capable of regulating the level of clustering inside a fruiting body. The 2D model simulations determined what patterns of spore clustering would emerge from cells aligned movement along slime trails and C-signaling by the end-to-end contact. In addition, the movement and interaction of cells in the 2D model included cell-cell and cell-spore collisions as well as cell reversals that reinforced alignment within the aggregate. We found that the coordinated movement of cells — by way of self-propelled motion, slime trail following, cell-cell and cell-spore collisions, and cell reversals — can facilitate the contact-dependent signal accumulation that drives cell differentiation into spores.

The integration of novel experimental observations with computational simulations provided new insight into the mechanisms that could give rise to the structure with a pattern of dense spore pockets seen during fruiting body formation. This can be improved upon through use of newer OCT devices with better resolution and even applied to other biological systems of cell aggregation such as that seen in dictyostelids, social amoeba known to form multicellular aggregates observed as slugs under starvation conditions.

Understanding how cells can undergo differentiation under specific spatial patterning is important to biology in general. It is known that chemical signals and reaction-diffusion processes can lead to coordination of cell patterning and differentiation. In the fruiting body process, we have shown how this patterning and differentiation could arise in the absence of a diffusive signal.

## Supporting Information

Movie S1 **Animation of 3D internal structure.** In the rotating animation, the isovalue is continuously increased in order to reveal the higher intensity (density) regions. Darker colors correspond to less intensity and lighter colors correspond to higher intensity. In the interior of the fruiting body, the higher intensity (more dense regions) form pockets or regions surrounded by less dense areas. A light gray transparent surface of the mound is persistently visualized in the animation.
(AVI)

Table S1 **Parameter values for 2D stochastic model.** This table contains the parameters, parameter values and descriptions for the parameters.
(EPS)
